# A robotic perturbation trainer for transverse-plane gait perturbations in pediatric cerebral palsy^,^

**DOI:** 10.1016/j.mex.2026.104051

**Published:** 2026-07-15

**Authors:** Amna R. Khawaja, Daulet Sagidoldin, Dilnoza Karibzhanova, Yinan Jin, Aibek Niyetkaliyev, Shyngys Dauletbayev, Naveed Ahmad Khan, Prashant K Jamwal

**Affiliations:** a Department of Physical Therapy, Athletic Training and Rehabilitation Science, School of Health Professions, University of Kansas Medical Center, Kansas City, KS, USA; b School of Engineering and Digital Sciences, Nazarbayev University, Astana, Kazakhstan; c Department of Mechanical Engineering, Zhejiang University of Technology, China; d Nazarbayev University Research Administration (NURA) Private Institution, Astana, Kazakhstan; e School of Information Technology and Systems, University of Canberra, Australia

**Keywords:** Cerebral palsy, Gait analysis, Pediatric rehabilitation, Perturbation-based training, Robot-aided rehabilitation

## Abstract

This study describes the development and implementation of a robotic perturbation-based training methodology for children with cerebral palsy (CP). A Robotic Perturbation Trainer (RPT) was engineered to deliver controlled multidirectional waist-pull perturbations during treadmill walking. The system integrates a cable-driven parallel manipulator with a treadmill and body-weight support to enable safe and repeatable application of external disturbances in the transverse plane.

The methodology was implemented in two adolescents with CP (ages 16–17; spastic diplegic and spastic dyskinetic subtypes) using a 5-week protocol consisting of ten treadmill-based sessions. Training parameters, including perturbation magnitude (65–85 N) and treadmill speed (0.2–0.5 km/h), were progressively adjusted according to predefined ranges. A multimodal assessment framework was applied at baseline, mid-, and post-implementation stages. Functional mobility and balance were evaluated using standardized outcome measures (6-Minute Walk Test, 10-Meter Walk Test, Timed Up and Go Test, Berg Balance Scale, and Gross Motor Function Measure), and surface electromyography was used to quantify activation of the gluteus maximus and medius muscles.

**The proposed methodology:**

Delivers controlled transverse-plane perturbations during treadmill walking using a cable-driven robotic system

Applies a structured, progressive perturbation training protocol under body-weight support conditions

Integrates clinical assessment tools with EMG-based muscle activity analysis for multimodal evaluation

## Graphical abstract

**Figure:**
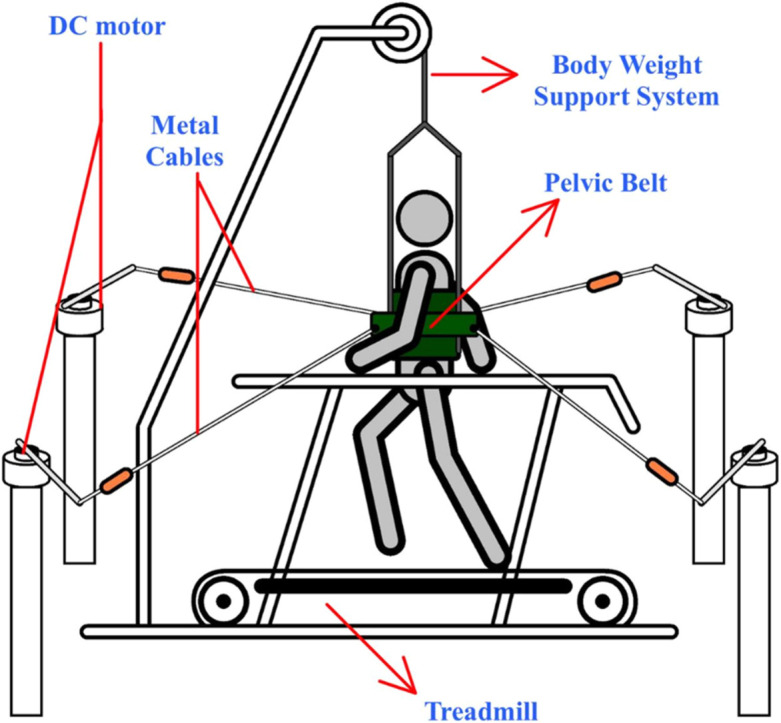

## Specifications table

**Table:** 

**Subject area**	Engineering
**More specific subject area**	*Biomedical engineering*
**Name of your method**	*A Robotic Perturbation-Based Training*
**Name and reference of original method**	*None*
**Resource availability**	*None*

## Background

Cerebral Palsy (CP) is a group of permanent movement disorders caused by injury to the central nervous system during the perinatal period [1]. CP affects around 2–4 children per 1000 live births [[2], [3], [4]] and represents a major pediatric condition requiring rehabilitation strategies to address motor impairments and functional limitations [5,6]. Postural control impairments are commonly faced among both children and adults, who have either mild or severe levels of CP, particularly in those diagnosed with spastic dyskinetic and spastic diplegic forms [[7], [8], [9]]. Spastic dyskinetic CP is characterized by involuntary, fluctuating movements, including dystonia and choreoathetosis, that affect motor coordination and stability [[10], [11], [12]]. Conversely, spastic diplegic CP primarily affects the lower body, causing muscle stiffness and making walking difficult. Individuals affected by this condition tend to have scissors-like movements during leg motion because the inflamed hip and thigh muscles pull the legs towards each other and cross at the knees [[13], [14], [15]]. These two types greatly impair balance and coordination; therefore, rehabilitation measures to address each must be specific to the motor issues associated with their respective types of CP.

Postural control is fundamental to maintaining balance and involves the coordinated integration of the vestibular, visual, proprioceptive, and motor systems. Impairments in these systems reduce stability and functional performance. Conventional therapies often fail to adequately challenge balance systems under realistic perturbations, motivating exploration of advanced interventions that can replicate real-world instability [16].

Perturbation-based training is one such approach, designed to expose individuals to controlled, unexpected disturbances during tasks to enhance reactive balance control and their ability to maintain or regain stability [17]. Previous studies have applied perturbations across various populations and contexts, including individuals with neurological conditions, using different modalities and perturbation directions [14,[18], [19], [20]].

In pediatric CP, robotic-assisted and treadmill-based interventions incorporating perturbations have been explored using different mechanical setups and planes of motion [21,22]. Repeated perturbations have been shown to be essential for leveraging residual learning abilities in patients with cerebellar ataxia, thereby improving balance control during steady locomotion [23]. Gradual perturbation impulses during treadmill training have been introduced as a strategy to assess retention of motor adaptations in children with cerebral palsy [24]. Frontal-plane perturbation using a Tethered Pelvic Assist Device was found to improve pelvic motion not only in the frontal plane but also in the sagittal and transverse planes, while increasing activation of the left gluteus medius muscle, which plays a key role in pelvic control and stability [25].

However, existing implementations are often limited to specific perturbation directions, stationary conditions, or non-standardized delivery mechanisms. In particular, the application of controlled transverse-plane perturbations during dynamic gait, along with simultaneous measurement of muscle activation patterns, remains insufficiently described.

To address this, the present work focuses on the development and methodological description of a Robotic Perturbation Trainer (RPT), a cable-driven robotic system capable of delivering safe, randomized waist-pull perturbations in the transverse plane, with broader applicability for neurological rehabilitation, fall-prevention training in the elderly, and even athletic performance enhancement.

## Method details

### Robotic perturbation trainer (RPT)

**Fig. 1:**
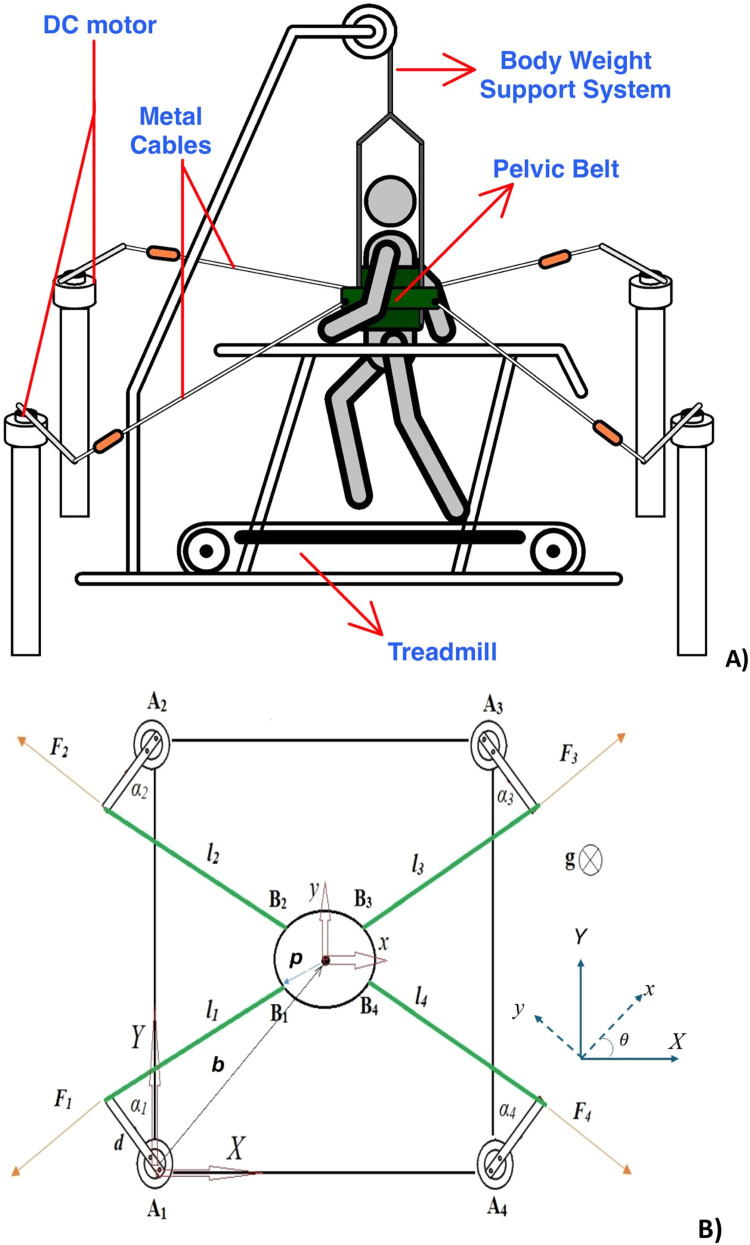
(A) Schematic representation of the Robotic Perturbation Trainer (RPT) integrated with a treadmill and body-weight support system. The setup includes four DC motor-driven actuators positioned at the treadmill corners; each connected via metal cables to a pelvic belt worn by the subject. (B) Kinematic Model of the Cable-Driven Planar Parallel Manipulator (CDPPM) (Top view) showing actuator positions (±45°, ±135°), arm length (0.25 m), cable routing ($l_{1}..l_{4}$), end-effector attachment points ($B_{1}..B_{4}$), and force directions ($F_{1}..F_{4}$), in the global XY and local *xy* coordinate frames.

To enhance dynamic balance through training with unexpected transverse perturbations, the RPT was conceptualized and developed for subjects walking on a treadmill. The system was designed and built to apply controlled, multi-directional external forces to the waist of a walking subject, as shown in Fig. 1(A). This configuration is intended to facilitate balance training and postural adaptation, particularly for individuals with neuromotor impairments such as CP. The RPT integrates seamlessly with a body-weight support system (BWSS) and a treadmill, ensuring both safety and repeatability in experimental and clinical training protocols. The mechanical structure consists of four rotary actuating units located slightly above the subject’s hip level, evenly distributed at the four corners of the treadmill at angular positions of approximately $\pm 45^{o}$ and $\pm 135^{o}$. Each actuating unit is based on a brushless DC motor, specifically the CubeMars AK80–64, capable of delivering up to 120 Nm of torque, which, given the actuator arm geometry, can generate a cable force of up to 480 N. The motors drive constant-length arms of 0.25 m each, routing a tensioned steel cable through directional pulleys to the waist-worn harness that serves as the end-effector (EE), as shown in Fig. 1(B).

By precisely controlling the torque at each motor, the system modulates the tensile forces $F_{1}$ through $F_{4}$ in the four cables, producing a resultant net force vector on the subject’s center of mass (CoM). The controller maintains a minimum baseline tension (10% of the maximum perturbation force) across all cables to keep them taut and superimposes perturbation forces according to a predefined protocol. Pretension is a necessary condition for controllability in all cable-driven robots [26]. The perturbation force is dynamically increased over the sessions, starting at 10% of the subjects’ weight and increasing to an upper bound of 85 N, while carefully monitoring subjects’ endurance and comfort. The driven cables are attached to four connection points on the end-effector (EE), a circular belt worn by the subject. This setup enables diagonal pulling motions. The four actuators in this CDPPM mechanism are controlled simultaneously to deliver precise, controlled perturbations during treadmill walking sessions, thereby enhancing the rehabilitation process for children with cerebral palsy (CP). The controller operates in torque-control mode, using motor current to regulate the applied force on each cable with high precision. This allows setting any desired force on any motor, enabling simulation of disturbances akin to those encountered in real-world walking environments.

Moreover, safety measures are embedded at multiple levels: hardware torque limits, maximum cable tension constraints, velocity and acceleration bounds, and real-time monitoring of all actuator states. The BWSS prevents falls during trials, while software safeguards ensure that perturbations remain within safe thresholds throughout the intervention. The combined mechanical and control architecture supports safe, repeatable, and customizable perturbations, enabling both research and therapeutic applications in reactive balance, motor learning, and rehabilitation for populations with impaired postural control.

The controller for the RPT generates precise yet unpredictable perturbations in the horizontal plane while maintaining constant cable pretension and enforcing actuator safety limits. It manages high-level event scheduling, perturbation magnitude and timing selection, and low-level motor control through a deterministic pipeline that includes a perturbation scheduler, tension and torque computation based on the system kinematic model, and real-time motor current regulation. This architecture ensures that each perturbation is delivered within predefined safety bounds and that the applied force is traceable from the intended vector to the resulting actuator commands. The RPT introduces several distinctive design and technical contributions. From a design perspective, it employs a cable-driven planar parallel manipulator (CDPPM) with four diagonally oriented cables that enable randomized transverse plane waist pull perturbations, a direction of disturbance that has not been explicitly explored in rehabilitation robots. From a technical perspective, the system applies a least squares-based tension distribution method that ensures static equilibrium while embedding explicit upper and lower tension bounds appropriate for pediatric use. This provides stable and safe force control while overcoming limitations commonly encountered in cable-driven manipulators. The controller integrates a deterministic perturbation scheduler with a pseudorandom event generator so that the timing and direction of perturbations cannot be anticipated by the subject, thereby enhancing the challenge to postural control. In addition, the RPT combines adaptive pretension management with progressive perturbation scaling from 65 to 85 Newtons, thereby allowing training intensity to be gradually increased in line with the subject’s endurance. Finally, the integration of the RPT with a treadmill and a body weight support system provides a compact and clinically applicable platform specifically suited for pediatric cerebral palsy rehabilitation, where safety, repeatability, and adaptability are critical requirements.

### Kinematic model of the robotic perturbation trainer (RPT)

The RPT is modelled as a 3-DOF planar cable-driven parallel manipulator. The rectangular treadmill frame serves as the global reference, with the four motor pivots fixed at its corners, $A_{i}$ (i=1,…,4). Each pivot carries a rigid arm of constant length d whose rotation angle is $\alpha_{i}$; the cable leaves the arm tip and attaches to the waist belt. The belt is treated as a rigid ring of radius p with a local frame at its centre and pose (x,y,θ). The four attachment points *(*$B_{1}..B_{4}$*),* have fixed local offsets $r_{i}=\left(r_{ix},r_{iy}\right)$, placed at $90^{\circ }$ intervals: $r_{1}=\left(p,0\right)$, $r_{2}=\left(0,p\right)$, $r_{3}=\left(-p,0\right)$, and $r_{4}=\left(0,-p\right)$. The global position of each belt attachment point is obtained by rotating the local offset by θ and translating by $b={\left(x,y\right)}^{T}$:

(1) $$B_{i}=b+R\left(\theta \right)\;r_{i}=\left(x+r_{ix}\text{cos}\theta -r_{iy}\text{sin}\theta ,y+r_{ix}\text{sin}\theta +r_{iy}\text{cos}\theta \right)$$

In this mechanism the cable is of fixed physical length; perturbation is generated by rotating the actuator arm, which displaces the cable anchor point ($A_{i}$) and thereby changes the effective taut span $l_{i}$ between the anchor and the belt attachment point $B_{i}$. Throughout this subsection, $l_{i}$ denotes this effective cable length.

The cable anchor at the arm tip moves on a circle of radius d about its pivot:

(2) $$A_{i}{}^{\prime }\left(\alpha_{i}\right)=A_{i}+d\;\left(\text{cos}\alpha_{i},\text{sin}\alpha_{i}\right)$$

So, the instantaneous cable lengths are the distances between their respective anchor points and the attachment points:

(3) $$l_{i}=∥B_{i}-A_{i}{}^{\prime }\left(\alpha_{i}\right)∥$$

#### Inverse kinematics (pose to motor angle)

For a desired belt pose, the arm angle that yields a target cable length follows from the law of cosines. Defining the vector from the pivot directly to the belt point, $R_{i}=B_{i}-A_{i}$, its length $L_{i}=∥R_{i}∥$, and its direction $\beta_{i}=\text{atan}2\left(R_{iy},R_{ix}\right)$:

(4) $$l_{i}^{2}=L_{i}^{2}+d^{2}-2\;d\;L_{i}\text{cos}\left(\alpha_{i}-\beta_{i}\right)$$

(5) $$\alpha_{i}=\beta_{i}+\text{arccos}\left(\frac{L_{i}^{2}+d^{2}-l_{i}^{2}}{2\;d\;L_{i}}\right)$$

Eq. (5) is the core inverse-kinematic relation that returns the four motor angles that produce a commanded change in cable lengths, and hence the desired end-effector pose.

#### Differential kinematics

Relating the pose and arm-angle rates to the cable-length rates gives $\dot{l}=J\;\dot{q}$, with the generalized coordinates $q={\left[x,y,\theta ,\alpha_{1},\alpha_{2},\alpha_{3},\alpha_{4}\right]}^{T}$ and the unit cable directions $u_{i}=\left(B_{i}-A_{i}{}^{\prime }\right)/l_{i}$. Row i of the 4×7 Jacobian (four rows for cable lengths and seven columns from the generalized coordinates q) is

(6) $$J_{i}=\left[\;u_{ix},u_{iy},u_{ix}\left(-r_{ix}\text{sin}\theta -r_{iy}\text{cos}\theta \right)+u_{iy}\left(r_{ix}\text{cos}\theta -r_{iy}\text{sin}\theta \right),\ldots ,d\;\left(u_{ix}\text{sin}\alpha_{i}-u_{iy}\text{cos}\alpha_{i}\right),\ldots \;\right]$$

where the only non-zero arm-angle entry is the one matching cable i, or in other words, each cable's length is affected by only its own arm motor, not the other three. The unit cable directions $u_{i}$ defined here are employed directly into the structure matrix A and the least-squares tension-distribution solution given in the following *Tension Distribution and Dynamics* subsection.

### Tension distribution

The RPT generates perturbations through the cable-driven planar parallel manipulator described above, in which four diagonally oriented cables apply controlled forces to the waist-mounted belt. Using the unit cable directions $u_{i}=\left(u_{ix},u_{iy}\right)$ and the belt attachment offsets $r_{i}$ defined in the kinematic model, the wrench exerted on the belt is related directly to the cable tensions.

For the manipulator to remain controllable, each cable must sustain a minimum pretension to avoid slackness, while respecting the maximum permissible force set by the safety requirements of the protocol:

(7) $$f_{min}\leq f_{i}\leq f_{max}$$

Here $f_{i}$ is the tension force in each cable. When the subject experiences a horizontal perturbation $F={\left[F_{x},F_{y},M_{z}\right]}^{T}$ at the waist, the four cable tensions $f={\left[f_{1},f_{2},f_{3},f_{4}\right]}^{T}$ must balance it in the transverse plane. Each cable contributes a planar force along its unit direction $u_{i}$ and a moment about the belt centre equal to ${\left(r_{i}\times u_{i}\right)}_{z}=r_{ix}u_{iy}-r_{iy}u_{ix}$. Collecting these contributions for the four cables yields the structure matrix $A\in R^{3\;\times \;4}$:

(8) $$A=\left[\begin{array}{cccc} u_{1x} & u_{2x} & u_{3x} & u_{4x}\\ u_{1y} & u_{2y} & u_{3y} & u_{4y}\\ {\left(r_{1}\times u_{1}\right)}_{z} & {\left(r_{2}\times u_{2}\right)}_{z} & {\left(r_{3}\times u_{3}\right)}_{z} & {\left(r_{4}\times u_{4}\right)}_{z} \end{array}\right]$$

For the symmetric attachment geometry $r_{1}=\left(p,0\right)$, $r_{2}=\left(0,p\right)$, $r_{3}=\left(-p,0\right)$, $r_{4}=\left(0,-p\right)$, the moment row evaluates to $\left(p\;u_{1y},\;-p\;u_{2x},\;-p\;u_{3y},\;p\;u_{4x}\right)$.

Static equilibrium requires Af=F. Since the RPT is over-actuated (four cables but only three planar wrench components), infinitely many tension vectors satisfy a given perturbation. To obtain a unique, closed-form solution, the minimum-norm tension distribution is used, subject to the lower tension bound:

(9) $$\underset{f}{\text{min}}\;{∥f∥}_{2}^{2}\text{subject}\;\text{to}\;Af=F,\;f_{i}\geq f_{\text{min}}$$

Since ${∥f∥}_{2}^{2}=f^{T}f$, Eq. (3) is a quadratic program with a linear equality constraint and bound constraints. It is solved in MATLAB using the lsqlin function, with the coefficient matrix C=I(identity) and target vector d=0, and with Af=F and $f_{i}\geq f_{\text{min}}$ imposed as the equality and bound constraints. The routine returns the minimum-norm tension vector that produces the desired perturbation wrench while keeping every cable above its pretension threshold.

### Implementation of unexpected perturbations

Four CubeMars® AK80–64 brushless DC motors are employed to drive the RPT, delivering a rated torque of 48 Nm, with a peak torque of 120 Nm. These motors feature a planetary gear system with a reduction ratio of 64:1 and include a 14-bit onboard encoder. An Odrive 24 V V3.6 brushless DC motor controller enables real-time control implementation. The Odrive board is equipped with a DRV8301 Three Phase Pre-Driver IC and an STM32F4 microcontroller based on the Arm® Cortex®-M4 architecture. To investigate balance training using unexpected perturbations, a simple controller was designed to produce discrete perturbations from a single cable at a time. For simplicity, the robot is considered as a static force distributor, and a pseudorandom generator is used to select which of the four cables will deliver the next perturbation. The perturbation magnitude is a fixed, user‑defined constant. Before delivering any perturbations, each cable is pretensioned to 10 N to keep all cables taut. This prevents slack and gives the controller an initial tension margin.

At each perturbation event the controller randomly chooses one cable index k∈{1,2,3,4} from a discrete uniform distribution using Mersenne Twister pseudorandom number generator. The random number is seeded once at the start of the experiment and used thereafter to pick the cable index. This ensures that subjects cannot anticipate which direction the next perturbation will come from. A constant value $F_{\text{pert}}$ within the desired range (65–85 N) is selected which is adjusted manually between trials to scale the task difficulty but remains fixed within a trial. The desired horizontal force vector is given by $F_{\text{des}}=F_{\text{pert}}e_{k}$, where $e_{k}$ is a unit vector aligned with $k^{th}$ cable’s pull direction.

The tension vector $f={\left[f_{1},\;f_{2},\;f_{3},f_{4}\right]}^{T}$ is updated so that only the selected cable delivers the perturbation:

(10) $$f_{i}=\left\{ \begin{array}{c} f_{pre}+f_{\text{pert}},\;if\;i=k\\ f_{pre}\;\text{otherwise} \end{array}\right.$$

The other three cables remain at the pretension level. Because each cable has a known pull direction in the transverse plane, this change produces a horizontal perturbation of magnitude *F*_pert_ acting on the belt in the chosen direction. The commanded tension *f_k_* is converted into motor torque via the arm (*T_k_*=*df_k_*) and sent to the actuator while the remaining motors are held at their pretension torques. After a pulse duration of 400 *ms*, the controller returns all four cables to the pretension level and waits for a randomly determined inter‑perturbation interval before triggering the next event. Because only one cable delivers the perturbation, the net torque about the belt is negligible, and the subject experiences a pure translational unexpected pull.

The Odrive multiplies the required motor torque ($T_{\text{motor}}=T_{k}/64$, for a gear ratio of 64:1) by the reciprocal of the motor torque constant (known $k_{T}$ for the motor in newton‑meters per ampere) to compute the phase‑current reference ($I_{ref}=T_{\text{motor}}/k_{T}$). This converts a mechanical torque into an electrical current signal. Finally, Odrive regulates the motor currents so that the electromagnetic torque matches $T_{\text{motor}}$. Throughout the motion the Odrive monitors the encoder and currents to maintain control accuracy and protects the system by enforcing limits on current, velocity and position.

## Bench validation of the actuation system

The study was approved by the Nazarbayev University Institutional Review Ethics Committee Board (IRB No:1081/21,052,025). Informed Consent was obtained from all participants and their legal guardians before participation in the study.

## Ethical approval

The study was approved by the Nazarbayev University Institutional Review Ethics Committee Board (IRB No:1081/21,052,025). Informed Consent was obtained from all participants and their legal guardians before participation in the study.

## Study population

A convenience sample of two adolescent male participants with cerebral palsy was recruited for this study. Participant (AB-16) was 16 years old, weighed 67 kg, and was 165 cm tall. He was diagnosed with spastic dyskinetic cerebral palsy, classified at Gross Motor Function Classification System (GMFCS) level III. He used a wheeled walker for mobility and did not wear orthotic devices during the intervention. Participant (AR-17) was 17 years old, weighed 45 kg, and had a height of 163 cm. He was diagnosed with spastic diplegic cerebral palsy, classified at GMFCS level I. He was independently ambulatory and wore an ankle foot orthosis (AFO) during the intervention. Both participants were cognitively intact, able to follow instructions, and free of recent orthopedic surgeries or medical conditions that could compromise safety during treadmill walking with perturbations. The details of the participants can be found in Table 1.

**Table 1:** Participants Details.

**Participant ID**	AB-16	AR-17
**Age (years)**	16	17
**Gender**	Male	Male
**Weight (kg)**	67	45
**Height (cm)**	165	163
**CP type**	Spastic Dyskinetic	Spastic Diplegic
**GMFCs Levels**	III	I
**Assisted Device**	Wheeled-Walker	None
**Orthotic Device**	AFO during intervention	None
**Ambulatory Status**	Assisted	Independent

**Inclusion Criteria:**

Participants were recruited for the study based on the following criteria:

• Age between 12–18 years.
• GMFCS level I-III.
• Cognitively intact.
• Can report pain.
• The upper limb should have a strength of +3 on Manual Muscle Testing.
• A score of ≤2 on Ashworth Scale.
• Absence of consolidated fractures and contracture.
• General Good Health.
• Height should be between 160 and 195 cm.
• The shoe size should be ≥38.

**Exclusion Criteria:**

Participants were excluded from the study based on the following criteria:

• Children not diagnosed with CP.
• Age below 12 or above 18 years.
• GMFCS level IV or V.
• With mental problems and cognitive impairment.
• Suffering from systemic diseases such as infectious diseases, circulatory problems, and heart or lung problems.
• Orthopedic Surgeries in the past 6 months.
• Severe spasticity (>2 on the Ashworth Scale).
• Heterotrophic Ossification that reduces ROM.
• Spinal or pelvic instability.
• Unhealed fracture of ASIS or PSIS.

### Participant recruitment

Participants were recruited using a structured screening process designed to ensure suitability for the perturbation-based training protocol. The inclusion criteria required a confirmed diagnosis of cerebral palsy (CP), the ability to walk on a treadmill with or without minimal assistance, adequate cognitive ability to follow verbal instructions, and no history of orthopedic surgery or botulinum toxin injections within the previous six months. Exclusion criteria included severe musculoskeletal deformities, uncontrolled epilepsy, cardiovascular instability, or any medical condition that could compromise safety during treadmill walking with perturbations.

### Intervention protocol

For each session, a perturbation was delivered every 15 to 18 *sec*., giving approximately 82–93 perturbations per session over 22- 24 mins of average active walking time of treadmill walking. This further means that the perturbation force was applied approximately 20–23 times in each direction per session. Magnitude was held constant within a trial and stepped up across sessions from the lower bound of the range.

### Experimental trials

**Fig. 2:**
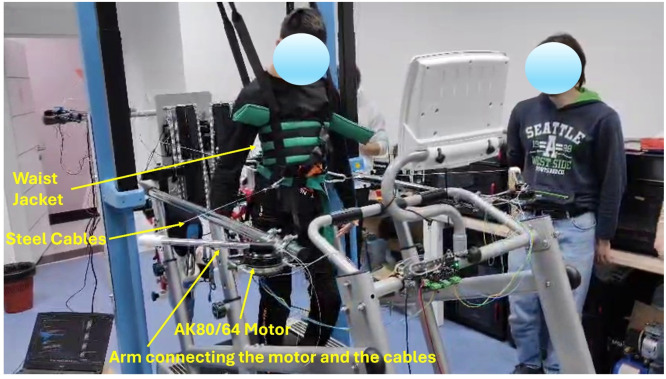
Experimental setup of the Robotic Perturbation Trainer, showing a participant with cerebral palsy during treadmill training, equipped with a waist jacket, steel cables, and AK80/64 motors to deliver controlled perturbations.

10 Training Sessions were conducted over 5 weeks, each lasting longer than 1.5 h, including subject preparation for trials, system calibration, and donning and doffing of the system. Before the training sessions began, one session was organized for information about the whole study participants and to obtain informed consent from subjects, and one session of assessment was conducted to record all clinical and biomechanical data before the intervention began, in the middle of the intervention (after 5 sessions), and after the intervention, in total 10 sessions of intervention were conducted for each subject. The perturbation system used in the study applied transverse plane forces at random to the subject’s waist from four different directions (front right & left, back right and left) while walking on the treadmill to investigate balance responses. The study participants wore a body-waist attached to BWSS, which prevented participants from falling during the training; however, the support from BWSS was always kept at a minimum (around 15–10%) of the participants’ body weight. Fig. 2 shows the experimental setup with the CP subject.

### Statistical analysis

All clinical outcome measures (6MWT, 10MWT, TUGT, BBS, GMFM) were administered three times at each assessment phase (pre, mid, post) by the same physiotherapist under standardized conditions; values are reported as mean ± standard deviation (SD) across these three trials. Given the two-participant design, no inferential testing was applied to the clinical measures; instead, the absolute and percentage change between phases was computed and interpreted against published minimal detectable change (MDC) and minimal clinically important difference (MCID) values for cerebral palsy, a change being regarded as real only if it exceeds the MDC and clinically meaningful only if it exceeds the MCID. For the surface-EMG data, where each muscle's activation was sampled over many gait cycles per phase, the Friedman test was used to identify phase-dependent differences, followed by Wilcoxon signed-rank pairwise comparisons with Bonferroni adjustment (α = 0.017); given the sample size, these EMG results are interpreted as descriptive.

## Method validation

The proposed methodology was validated through bench validation of the actuation system and implementation in two adolescents with cerebral palsy (spastic diplegic and spastic dyskinetic subtypes) across three assessment phases (pre-, mid-, and post-intervention). Validation focused on system performance, protocol execution, and consistency of measurement outputs.

A. Device performance:

Toques delivered from the four motors were measured using motor phase currents (Odrive currents) and the torque constant provided by the supplier (force = current × k_T ÷ arm length, with arm length 0.25 m). To validate the measured perturbation forces in cables, four in-line load cells, LCM300 (250 lb) from FUTEK®, are installed in series with each cable to measure instantaneous cable tension in real time. Perturbations are randomly applied by applying a transient force pulse to one of the four cables, without employing any human participant.

• Force-tracking accuracy: Across commanded targets spanning the operating range (pretension 10 N and perturbations at 65–85 N), in each of the four pull directions, with 10 repetitions per target–direction combination, commanded versus measured cable force showed a mean absolute error of 2.3 N, a mean relative error of 3.1%, and an RMSE of 2.8 N.
• Consistency/repeatability: The within-target standard deviation of delivered force was ≤ 1.6 N across repetitions and ≤ 2.0 N across the four directions, indicating direction-independent delivery.
• Pretension maintenance: Baseline tension was held at 10.2 ± 0.7 N per cable over a representative session, with a maximum observed drift of 1.4 N.
• Pulse dynamics / return to baseline after perturbation peak force: For the 400 ms perturbation pulse, the actuator reached the required commanded peak force in the interval of 65–85 N with a rise time of around 100 ms and an overshoot of <6%. Following pulse termination, the cable force returned to the pretension level within approximately 120 ms.

B. Clinical Outcomes Progression Over Time:

Table 2 summarizes training parameters for each participant (AB-16 and AR-17) averaged over 10 sessions. Table 3, Table 4 present the phase-wise results for AR-17 (spastic diplegic CP) and AB-16 (spastic dyskinetic CP). Table 3, Table 4 present, for each measure, the mean ± SD at each phase (across three trials), the absolute and percentage change from pre to post, and the corresponding MDC and MCID with their interpretation.

1) For AR-17:

**Table 2:** Summary of training parameters for each participant (AB-16 and AR-17) averaged over ten sessions.

**Participants**	**Av. Treadmill speed (km/hr.)**	**Av. Active walking time (min)**	**Av. Perturbation magnitude (N)**	**Av. Total perturbations**	**Av. Perturbations per direction**	**Av. BWSS %**
***AB-16***	0.39	22	68	82	∼20	12.5
***AR-17***	0.42	24	73	93	∼23	12.33

**Table 3:** Clinical metrics across intervention phases for AR-17 (spastic diplegic CP): mean ± SD at each phase (three trials), pre-to-post change, and interpretation against MCID.

**Metric**	***Pre (mean ± SD)***	***Mid (mean ± SD)***	***Post (mean ± SD)***	***Δ Pre→Post (abs, %)***	***MCID***	***Interpretation***
***6MWT (m)***	181 ± 74.95	238.5 ± 6.36	185.5 ± 81.31	+4.5 (+2.5%)	6–23 m	Improvement observed; did not reach the lower MCID threshold
***10MWT (s)***	15.79 ± 3.55	13.09 ± 0.26	15.60 ± 3.82	−0.19 (−1.2%)	Not established for pediatric CP	Improvement observed; clinical significance cannot be determined
***TUGT (s)***	17.31 ± 2.38	14.61 ± 1.43	16.30 ± 3.81	−1.01 (−5.8%)	Not established for pediatric CP	Improvement observed; clinical significance cannot be determined
***BBS (pts)***	48.5 ± 2.12	50.5 ± 0.70	49.0 ± 2.82	+0.5 (+1.0%)	Not established for pediatric COP	Improvement observed; clinical significance cannot be determined
***GMFM (%)***	86.35 ± 4.03	89.4 ± 0.288	86.55 ± 4.31	+0.2 (+0.2%)	0.1–3.0%	Changes fall within the reported MCID ranges

**Table 4:** Clinical metrics across intervention phases for AB-16 (spastic dyskinetic CP): mean ± SD at each phase (three trials), pre-to-post change, and interpretation against MDC and MCID.

***Metric***	***Pre (mean ± SD)***	***Mid (mean ± SD)***	***Post (mean ± SD)***	***Δ Pre→Post (abs, %)***	***MCID***	***Interpretation***
***6MWT (m)***	220 ± 56.56	284.5 ± 34.64	244.5 ± 91.21	+24.5 (+11.1%)	6–23 m	Improvement exceeded the upper MCID threshold
***10MWT (s)***	13.85 ± 0.77	13.2 ± 1.69	12.65 ± 0.91	−1.2 (−8.7%)	Not established for pediatric CP	Improvement observed; clinical significance cannot be determined
***TUGT (s)***	27.5 ± 10.60	17.5 ± 3.53	25.0 ± 14.14	−2.5 (−9.1%)	Not established for pediatric CP	Improvement observed; clinical significance cannot be determined
***BBS (pts)***	33.5 ± 3.53	38.5 ± 3.53	36.0 ± 7.07	+2.5 (+7.5%)	Not established for pediatric CP	Improvement observed; clinical significance cannot be determined
***GMFM (%)***	66.38 ± 14.16	78.2 ± 2.54	68.19 ± 16.70	+1.81 (+2.7%)	0.1–3.0%	Changes fall within the reported MCID range

Note: MCID values for the 6-Minute Walk Test (6MWT) and Gross Motor Function Measures (GMFM-88) were obtained from published data [27].

For AR-17, 6MWT values were recorded as 181 ± 74.95 m (Pre), 238.5 ± 6.36 m (Mid), and 185.5 ± 81.31 m (Post). The 10MWT values were 15.79 ± 3.55 s (Pre), 13.09 ± 0.26 s (Mid), and 15.60 ± 3.82 s (Post), while TUGT values were 17.31 ± 2.38 s, 14.61 ± 1.43 s, and 16.3 ± 3.81 s across the respective phases. BBS scores were 48.5 ± 2.12, 50.5 ± 0.70, and 49 ± 2.82, and GMFM scores were 86.35 ± 4.03, 89.4 ± 0.28, and 86.55 ± 4.31. Relative to the published MCID for cerebral palsy, the pre-to-post changes are interpreted in Table 3; for AR-17, the largest gains occurred at the mid-point, with values regressing toward baseline at post.

2) For AB-16:

For AB-16, 6MWT values were 220 ± 56.56 m (Pre), 284.5 ± 34.64 m (Mid), and 244.5 ± 91.21 m (Post). The 10MWT values were 13.85 ± 0.77 s, 13.2 ± 1.69 s, and 12.65 ± 0.91 s, while TUGT values were 27.5 ± 10.60 s, 17.5 ± 3.53 s, and 25 ± 14.14 s across phases. BBS scores were 33.5 ± 3.53, 38.5 ± 3.53, and 36 ± 7.07, and GMFM scores were 66.38 ± 14.16, 78.2 ± 2.54, and 68.18 ± 16.70. Relative to the published MCID for cerebral palsy, the pre-to-post changes are interpreted in Table 4; AB-16 showed the larger functional gains of the two participants.

**Fig. 3:**
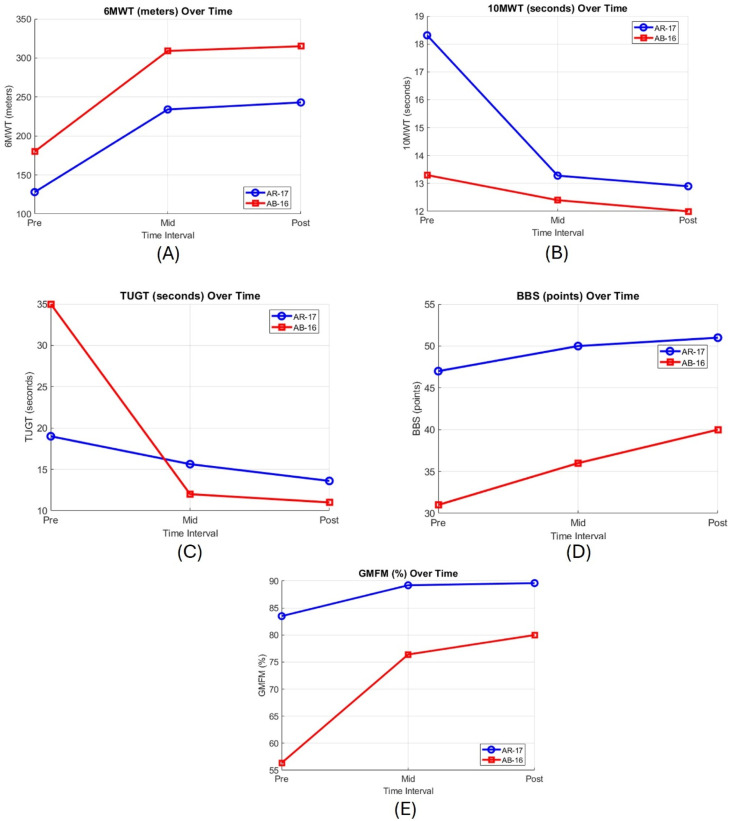
Progression of Functional and Clinical Outcomes Over Time (A: 6MWT, B: 10MWT, C: BBS, D: TUGT, E: GMFM) Following Perturbation-Based Training in Subjects with Cerebral Palsy.

B. Intervention Parameters:

Training parameters were progressively adjusted across sessions for both participants. Treadmill speed ranged from 0.2 to 0.5 km/h, session duration increased from 10 to 23 min, and perturbation forces were applied within the range of 65 to 85 N. All sessions were completed according to the predefined protocol without interruption (Fig. 3).

C. Electromyographic measurements:

Surface EMG recordings of the gluteus maximus and gluteus medius muscles were obtained bilaterally across all phases. Statistical analysis using Friedman’s test identified phase-dependent differences in selected muscle activation variables (p < 0.005). Pairwise comparisons using the Wilcoxon signed-rank test with Bonferroni correction were performed across phases.

Table 5 summarizes the Friedman test results for both participants. Differences were identified in Gluteus Maximus (right and left) and Gluteus Medius (right) for both subjects (p < 0.005), while Gluteus Medius (left) showed variable results across participants.

**Table 5:** Friedman's Test Results for Gluteal Muscle Activation for both AB-16 & AR-17).

**Variables**	**χ(AR-17)**	**Prob>χ (AR-17)**	**χ(AB-16)**	**Prob>χ (AB-16)**
Glut Max (R)	22.06	p < 0.005	12.18	p < 0.005
Glut Max (L)	12.87	p < 0.005	25.1	p < 0.005
Glut Med (R)	83.07	p < 0.005	51.11	p < 0.005
Glut Med (L)	25.28	p < 0.005	3.43	p > 0.005

Given the small sample size, the EMG findings should be interpreted as exploratory observations intended to demonstrate protocol feasibility and characterize muscle activation responses, rather than to establish intervention efficacy.

D. Protocol execution:

All training sessions were completed under predefined conditions, including perturbation magnitude, treadmill speed, and safety constraints. No deviations from the protocol were recorded. Data collection was completed at all planned time points, and no missing measurements were observed.

## Limitations

The proposed methodology has several limitations. First, the protocol was implemented on a small number of participants, which may limit its generalizability to a broader CP population. Further application in larger and more diverse cohorts is required.

Second, the assessment focused on selected hip muscles using surface EMG, which may not capture the full range of neuromuscular responses during perturbation-based training. Future work may incorporate additional muscle groups and biomechanical measures for a more comprehensive evaluation.

## Ethics statements

The study was approved by the Nazarbayev University Institutional Review Ethics Committee Board (IRB No:1081/21,052,025). Informed Consent was obtained from all participants and their legal guardians before participation in the study.

## CRediT author statement

Amna R. Khawaja: Conceptualization, Methodology, Investigation, Writing – Original draft preparation.

Daulet Sagidoldin: Software, Hardware development, Data curation, Validation.

Dilnoza Karibzhanova: Methodology, Investigation, Clinical assessment, Writing – Reviewing and Editing.

Yinan Jin: Formal analysis, Data analysis, Visualization.

Aibek Niyetkaliyev: Hardware development, System integration, Validation.

Shyngys Dauletbayev: Software, Data acquisition, Technical support.

Naveed Ahmad Khan: Supervision, Writing – Reviewing and Editing.

Prashant K. Jamwal: Conceptualization, Supervision, Writing – Reviewing and Editing.

## Declaration of competing interest

The authors declare that they have no known competing financial interests or personal relationships that could have appeared to influence the work reported in this paper.

## Data Availability

Data will be made available on request.
